# Pathogenicity of entomopathogenic *Beauveria bassiana* strains on *Helicoverpa armigera* (Hübner)

**DOI:** 10.3389/finsc.2025.1552694

**Published:** 2025-04-11

**Authors:** Rachid Boulamtat, Karim El Fakhouri, Hassna Jaber, Ali Oubayoucef, Chaimae Ramdani, Nabil Fikraoui, Muamar Al-Jaboobi, Meryem El Fadil, Ilyass Maafa, Abdelhalem Mesfioui, Seid Ahmed Kemal, Mustapha El Bouhssini

**Affiliations:** ^1^ International Center for Agricultural Research in the Dry Areas (ICARDA), Rabat, Morocco; ^2^ AgroBioSciences Program, College of Agriculture and Environmental Sciences, Mohammed VI Polytechnic University, Ben Guerir, Morocco; ^3^ Biology Department, Faculty of Sciences, Mohammed V University, Rabat, Morocco; ^4^ Laboratory of Biology and Health, Department of Biology, Faculty of Science, Ibn-Tofail University, Kenitra, Morocco

**Keywords:** chickpea pod borer, *Beauveria bassiana*, virulence, endophyte, chickpea

## Abstract

The destructive pest of chickpeas, *Helicoverpa armigera* (Hübner), is difficult to control using synthetic insecticides. The current research examined the entomopathogenic and endophytic colonisation effects of three fungal strains of *Beauveria bassiana* (HASS; RFSL10; SP-IR-566) against *H. armigera* larvae under laboratory, greenhouse, and field conditions. Four inoculation methods were used in the greenhouse: Root Dipping (RD), Leaf Spraying (LS), Stem Injection (SI), and Seed Coating (SC), while spray application was used for laboratory and field treatments. Under laboratory conditions, the highest entomopathogenic effect was recorded by HASS and RFSL10 strains applied as a direct spray at 10^8^ conidia mL^-1^ with 100% mortality, followed by SP-IR-566 with 96%, 12 days after treatment. Furthermore, foliar application in the field reduced larval population by an average ranging from 82 to 100%, confirming the significant effects of the three tested strains. In terms of endophytic colonisation under greenhouse setting, both stem injection and root dipping methods expressed low to moderate mortality rates ranging from 32 to 40%, 15 days after application. These findings suggested that *B. bassiana* strains, investigated as foliar application, had a potential as an effective strategy to control *H. armigera.* This study also offers new insights into the potential of the endophytic entomopathogens approach as a viable and safe alternative to chemical pesticides.

## 1 Introduction

Climate and farming system changes contribute to the emergence of minor and/or new pests affecting food crops in different regions (1). Among these pests, chickpea pod borer (*Helicoverpa armigera*), is becoming an important pest in North African countries like Morocco (2, 3). The pest causes significant losses in crops such as chickpeas, cotton, tomatoes, sunflowers, beans, and maize. In Morocco, chickpea pod borer can reduce crop yield up to 31% (4). Currently, the most common method of managing the pest involves the extensive use of synthetic insecticides (4–6). As a result, *H. armigera* develops resistance to most synthetic insecticides (7, 8). The use of chemical pesticides poses environmental risks, as they contribute to pollution and undesirable side effects on beneficial organisms (9–11). To minimize the impact of excessive use of chemical insecticides to control *H. armigera*, it is crucial to develop components for Integrated Pest Management (IPM) for sustainable pest management practices (12, 13). Thus far, no reliable sources of resistance to pod borer exist in the primary chickpea gene pool to develop commercial varieties (14, 15). Recent studies have shown that plant extracts and entomopathogenic fungi can play key roles in the management of *H. armigera* (16–18). The entomopathogenic fungi have several physiological effects, including growth and oviposition perturbation, larval mortality, disruption of reproduction, and regulatory activities (19, 20).

*Beauveria bassiana* (Balsamo) Vuillemin (Hyphomycetes) is one of the most characterized and widely used entomopathogenic fungal species to manage polyphagous pests, such as chickpea pod borer (21, 22). Numerous *B. bassiana* based formulations are already commercialized in many countries against various agricultural pests (23–26). Besides its ability to parasitize insects, *B. bassiana* acts as an endophytic agent in several plant species and protects against pathogens (27–31). For example, *B. bassiana* was effective in controlling *Dactylopius opuntiae*, which devastates cactus plants in Morocco (26).

The present study aimed to validate the virulence of three *B. bassiana* strains in controlling *H. armigera* under laboratory and field conditions.

## 2 Materials and methods

### 2.1 Insect rearing

*Helicoverpa armigera* larvae were collected from infested chickpea fields between May and June 2021 at the ICARDA (International Center for Agricultural Research in the Dry Areas) experimental station in Marchouch, Morocco (33°56’10”N 6°69’21”W). The larvae were reared on an artificial diet according to Boulamtat et al. (17), under 25 ± 2°C temperature, 75 ± 5% relative humidity (RH), and 14:10 L/D photoperiod. Each larva was placed individually in a Petri dish and fed an artificial diet (32), until the pupation. After emergence, adults were maintained in glass cages (90 cm^3^ volume) with 10% honey solution as an energy source. After oviposition, eggs were collected daily and transferred into Petri dishes. The newly hatched larvae were reared on the artificial diet following the same procedure indicated above. The second instar larvae were used for various bioassays.

### 2.2 Entomopathogenic fungal strains

Three *B. bassiana* strains were collected from Syria (two strains) and Iran (one strain), originating from three insect hosts, and were used in this study (**Table 1**).

**Table 1:** *B. bassiana* strains, hosts, and collection sites.

Name	Collection sites	Host	Year of collection
HASS	Swidaa-Syria	Chickpea Pod borer (*Helicoverpa armigera*)	2010
RFSL10	Lattakia-Syria	*Red palm weevil* (*Rhynchophorus ferruginus*)	2012
SP-IR-566	Kohesabz, Fars-Iran	*SunPest* (*Eurygaster integriceps*)	2001

*B. bassiana* inoculum production: The three *B. bassiana* strains were multiplied on Potato Dextrose Agar medium (PDA) under 25 ± 1 C° temperature, and 14:10 L/D light cycle for 14 days. Spores were harvested by scraping the PDA plates with a sterile scalpel and suspended in 10 mL of sterile distilled water containing Tween-80 (0.01%).

### 2.3 Laboratory bioassay

All strains were tested on *H. armigera* larvae for their pathogenicity. Three conidial concentrations (10^6^, 10^7^, and 10^8^ conidia mL^-1^) were prepared in sterile distilled water containing Tween-80 (0.01%). Second instar larvae were inoculated by spraying 15 µL of the suspension using a sprayer with a 50 mL capacity, delivering a frequency of 1-2 mL per minute to ensure precise application. the conidia suspension on top of the insect. Five treated larvae were transferred into Petri dishes containing moist sterile filter paper and provided with an artificial diet. The bioassay was conducted using a completely randomized design with five replicates per concentration for each treatment and five larvae were used in each replication. Larvae treated with distilled water containing Tween-80 (0.01%) served as a control. The experiments were conducted under laboratory conditions at 24 ± 1°C temperature, 75 ± 5% RH, and 14:10 L/D cycle light. Larval mortality was recorded daily for 14 days. Mortality was calculated according to Abbott's formula (33):


$$\begin{array}{c} \text{Corrected Mortality }\left(\%\right)\\ =\frac{\left[\%\text{mortality in treatment}-\%\text{mortality in control}\right]}{\left[100-\%\text{mortality in control}\right]}\times 100 \end{array}$$

### 2.4 Greenhouse bioassay

Chickpea seedlings of Bouchra (cultivar FLIP97-114C) were inoculated with each strain at 10^8^ conidia mL^-1^ containing Tween-80 (0.01%). Four inoculation methods in five replications were evaluated. Each replication (pot) was planted with five seeds.

a) Root dipping (RD): Three-day-old germinated seeds were dipped in 10^8^ conidia mL^-1^ suspension containing Tween-80 (0.01%) for 90 min. Control seeds were soaked in a Tween-80 solution (0.01%).

b) Stem injection (SI): Twenty-day-old plants were individually injected at the stem and nodes with 1 mL of 10^8^ conidia mL^-1^ suspension using a sterile syringe. Control plants were injected by water plus Tween.

c) Fungi-sorghum seed inoculum (FS): Three-day-old germinated seeds were transplanted into a pot containing 2.5 g of sorghum grains pre-infected with the fungus strains. Control seeds were transplanted with 2.5 g sterile sorghum grains.

d) Leaf spray (LS): Twenty-day-old plants were sprayed with a fungal suspension of 1.10^8^ conidia mL^-1^ using a 500 mL hand-held plastic sprayer. Control plants were sprayed with water.

After inoculation, the plants’ pots were randomized and kept under greenhouse conditions at 22 ± 2°C temperature and 14:10 L/D cycle light and watered as needed.

#### 2.4.1 Fungi-sorghum seed inoculum preparation

The seed coating was prepared using sorghum seeds, following a modified method of Nankinga (34). Washed sorghum seeds (200 g) were subdivided into flasks, then boiled in 10 mL of distilled water and sterilized by autoclaving at 121°C at 1 bar for 15 min. After cooling, each flask received 10 mL suspension of 10^7^ conidia mL^-1^ for each strain, while the control flask received 10 mL of 0.01% (w/v) Tween-80 without the fungus. The flasks were incubated for four weeks in an incubator at 22 ± 2°C and were manually shaken every day. The final product was randomly distributed among the plant replicates.

#### 2.4.2 Larvicidal activity under greenhouse

Second instar larvae were fed with leaves randomly collected from plants of each inoculation method and the control. Larvae were considered dead if no movement was observed after lightly brushed. Dead larvae were removed and living larvae were kept until they pupated. Larvae mortality due to *B. bassiana* strains was confirmed from the mycosed larvae by placing them on Petri dishes lined with moistened filter paper and incubated for five days, then cultured on PDA and identified. Mortality was calculated according to Abbott’s formula (33).

### 2.5 Endophytic colonization

The presence of *B. bassiana* was evaluated using three separate treatments for each strain. Sections were cut from leaves and all selected plant parts. Then after, surfaces were sterilized with ethanol (70%) for 30 s, sodium hypochlorite (1.5%) for 90 s, and rinsed with sterilized water for 2 min. The sections were dried and placed on Petri dishes containing PDA medium. Fungal identity was confirmed using morphological techniques.

### 2.6 Field trials

The field trial was conducted in May 2022 at the ICARDA Marchouch Research Station, Morocco (33°56’10”N 6°69’21”W). The trial was conducted in a randomized complete block design with three replications using the chickpea variety Bouchra (FLIP97-114C). Each plot was planted with four rows of 1 meter length, and 0.6-meter spacing between rows. A 3-meter distance was kept between plots and blocks to prevent spray drift to adjacent plots. Normal agronomic practices were followed for growing the crop using a seeding rate of 100 kg/ha.

Three *B. bassiana* strains (10^8^ spores per mL), insecticide (emamectin benzoate at 250 g/ha), and control plots sprayed with water plus Tween-80 were applied at economic threshold (one larva per meter row) (35). The non-ionic surfactant Tween-80 (0.01%) was used as an emulsion to mix the spores with water before applying the treatments. The strains were sprayed twice with an interval of one week between the first and the second spray using a two-liter hand sprayer with a frequency of 8 mL/min. Larvae mortality was recorded at 1, 3, and 7 days after each spray application.

### 2.7 Data analysis

Mortality percentages were transformed into angular values (arcsine √P) before the statistical analyses for laboratory and greenhouse bioassays, and the transformed percentages were subjected to a two-way analysis of variance (ANOVA). The means were compared by Newman–Keuls tests at *p* < 0.05, with strains, concentration, exposure time, and method of inoculation as explanatory variables and percentage larval mortality as response variables. Under field conditions, the transformed percentages were subjected to a two-way analysis of variance. Using Henderson and Tilton’s (36) formula, the efficacy of the tested treatments was expressed as a percentage of reduction of *H. armigera* larvae number, as follows:


$$Reduction\%=\left(1-\frac{n\;in\;Co\;befor\;treatment\;\times n\;in\;T\;after\;treatment}{n\;in\;Co\;after\;treatment\;\times n\;in\;T\;before\;treatment}\right)\times 100$$

n: Insect population, T: treated, Co: Control

Probit analysis was used to calculate the lethal time (LT_50_) values for each strain, including their 95% confidence limits, using IBM SPSS Statistics 27.0. The computations were carried out using GenStat (22^nd^ Edition, VSN International, UK).

## 3 Results

### 3.1 Laboratory bioassays

#### 3.1.1 Pathogenicity of fungal strains on *H. armigera*

**Figure 1:**
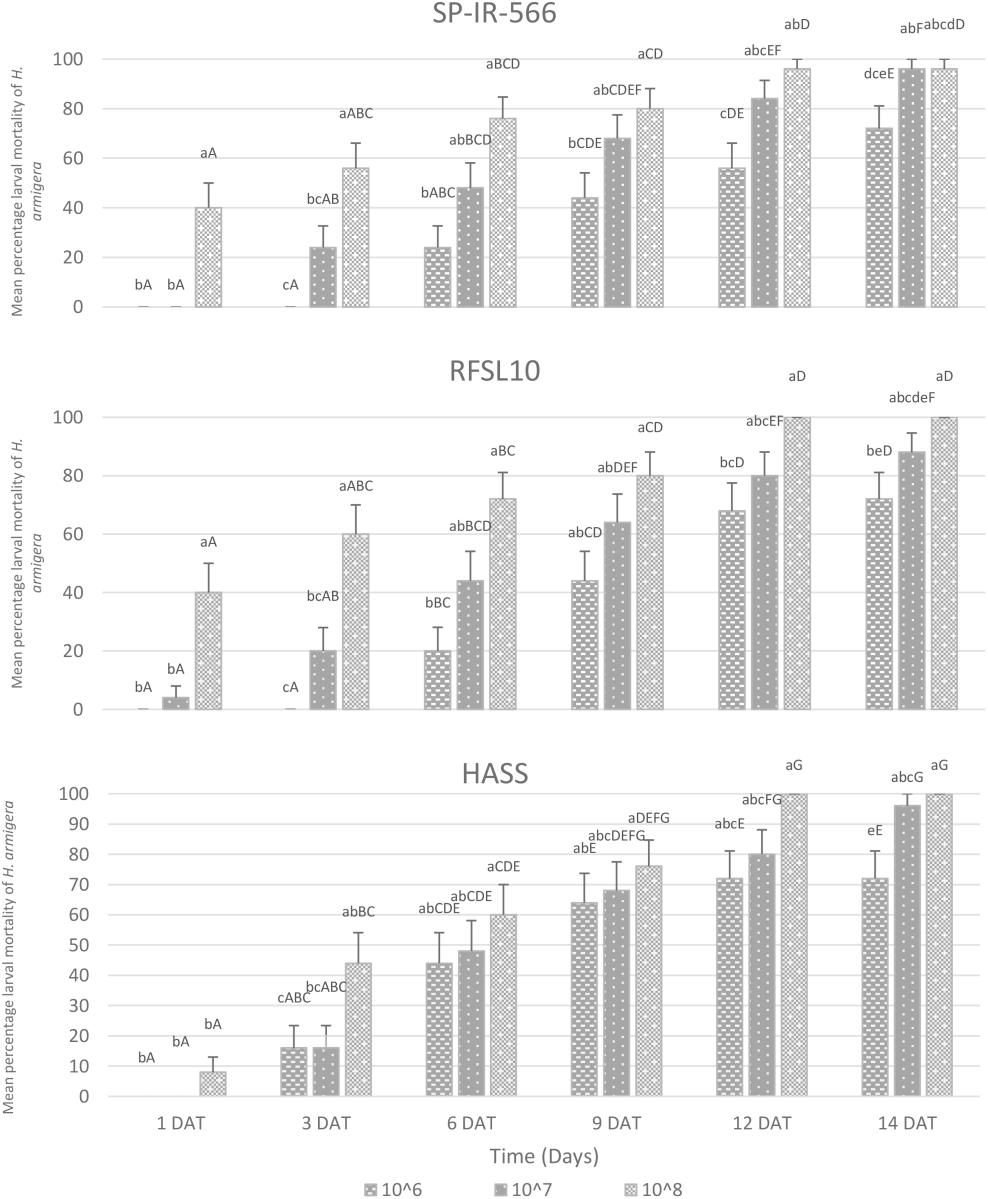
Mean percentage larval mortality of *H armigera* treatment with three *B bassiana* strains at three different concentrations under laboratory conditions, DAT, days after treatment. The means that are followed by the different lower-case letters indicate a significant difference among different treatment time, and different capital letters indicate a significant difference between different concentrations (p <0.001).

The mortality rates of *H. armigera* larvae after treatment with different strains of entomopathogenic *B. bassiana* are shown in **Figure 1**. All three *B. bassiana* strains were virulent to *H. armigera* larvae and their virulence varied significantly compared to the control (*p* < 0.001). Among the three tested strains, *B. bassiana* HASS and RFSL10 exhibited the highest virulence compared to SP-IR-566. Six days after treatment (DAT), the highest larval mortality was recorded by *B. bassiana* RFSL10 then *B. bassiana* RFSL10 with 76%, and 72%, respectively, at 10^8^ conidia mL^-1^. Twelve days after treatment, HASS and RFSL10 strains displayed the highest mortality rates (100% mortality), followed by strain SP-IR-566 with 96% mortality at 10^8^ conidia mL^-1^.

#### 3.1.2 Mean lethal time of strains

The mean lethal time (LT_50_) of all strains is presented in **Table 2**, based on the daily observation of *H. armigera* larvae mortality. All strains caused more than 50% mortality, demonstrating this fungus’ efficacity. The effects obtained with the strains RFSL10 and SP-IR-566 were significant at a concentration of 10^8^ conidia mL^-1^, with LT_50_ of 2 days. Furthermore, the LT_50_ was 6 days at a concentration of 10^7^ conidia mL^-1^. However, the LT_50_ for the HASS strain was 4 days at a concentration of 10^8^ conidia mL^-1^ and 7 days at 10^7^ conidia mL^-1^. No dead insects were observed in the control group.

**Table 2:** Mean lethal time (LT_50_) and confidence limit values of three *B. bassiana* strains at three concentrations against *H. armigera* larvae under Laboratory conditions.

*B. bassiana* strains	Concentrations (conidia mL^-1^)	LT_50_	95% confidence limit		Coefficient of determination (R²)	Graphing linear equations
			Lower limit	Upper limit		
HASS	10*^6^*	10.02	9.1	11.16	R² = 0.9383	y = 0.136x − 0.192
	10*^7^*	7.13	6.45	7.77	R² = 0.93	y = 0.148x − 0.228
	10*^8^*	4.13	3.53	4.69	R² = 0.9964	y = 0.0815x − 0.0797
RFSL10	10*^6^*	9.27	8.51	10.11	R² = 0.9944	y = 0.184x − 0.16
	10*^7^*	6.26	5.48	7.06	R² = 0.989	y = 0.152x − 0.096
	10*^8^*	2.06	1.43	2.65	R² = 0.98	y = 0.,088x − 0.0738
SP-IR-566	10*^6^*	7.2	6.39	8.07	R² = 0.8538	y = 0.1x + 0.356
	10*^7^*	6	5.34	6.64	R² = 0.9235	y = 0.104x + 0.352
	10*^8^*	2.05	1.34	2.71	R² = 0.894	y = 0.07x + 0.123

*LT50*: Lethal time for 50% mortality. *R* ^2^: Coefficient of determination. *Conidia mL^-1^*: Concentration of fungal spores (conidia) per milliliter.

### 3.2 Greenhouse bioassay

#### 3.2.1 Virulence of fungal strains on *H. armigera*

**Figure 2:**
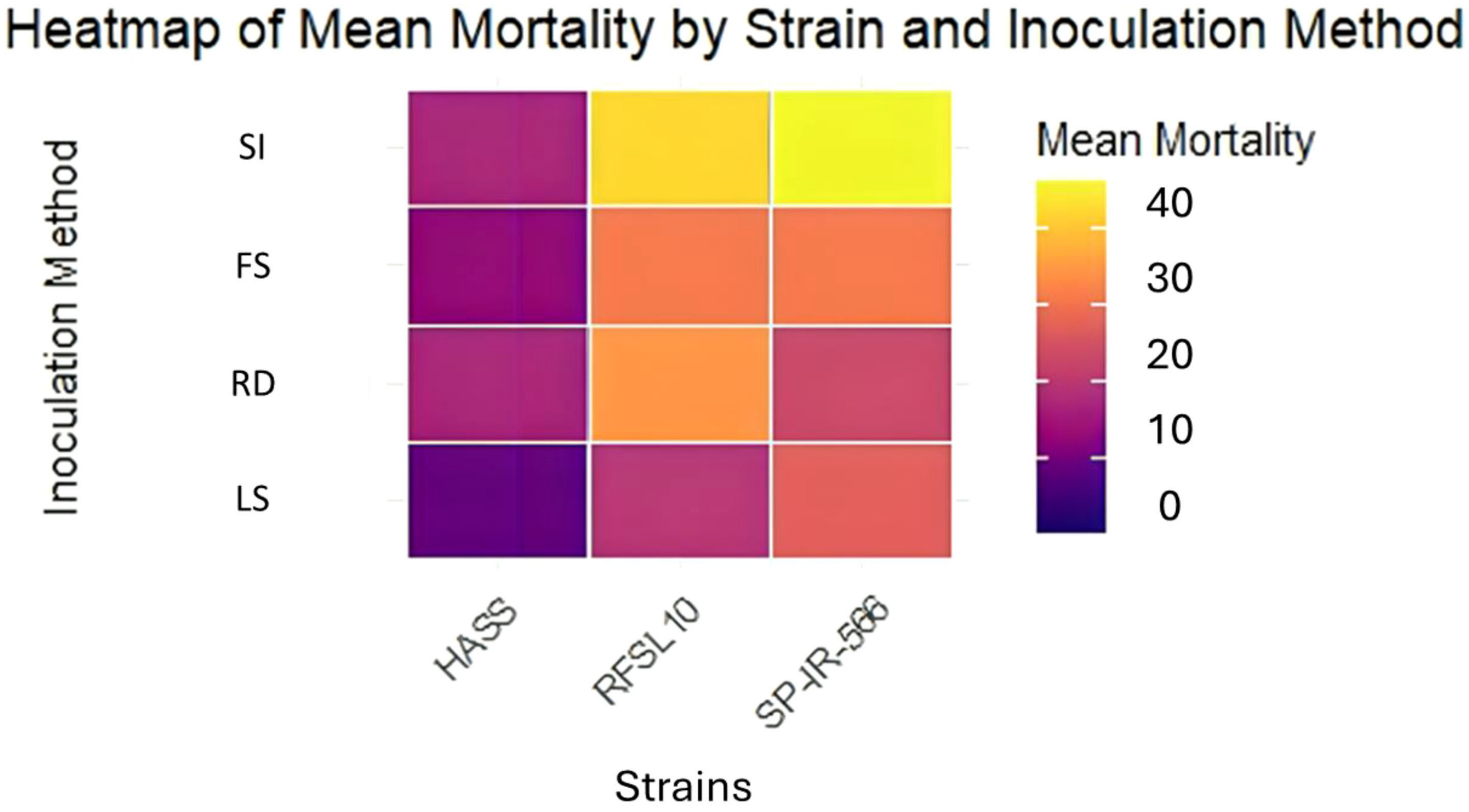
Heatmap representing the endophytic activity of the three strains against the larvae of *H. armigera* using different inoculation methods under greenhouse conditions. SI, stem injection; FS, fungi-sorghum seed inoculum; RD, root dipping; LS, leaf spray under greenhouse conditions.

**Figure 3:**
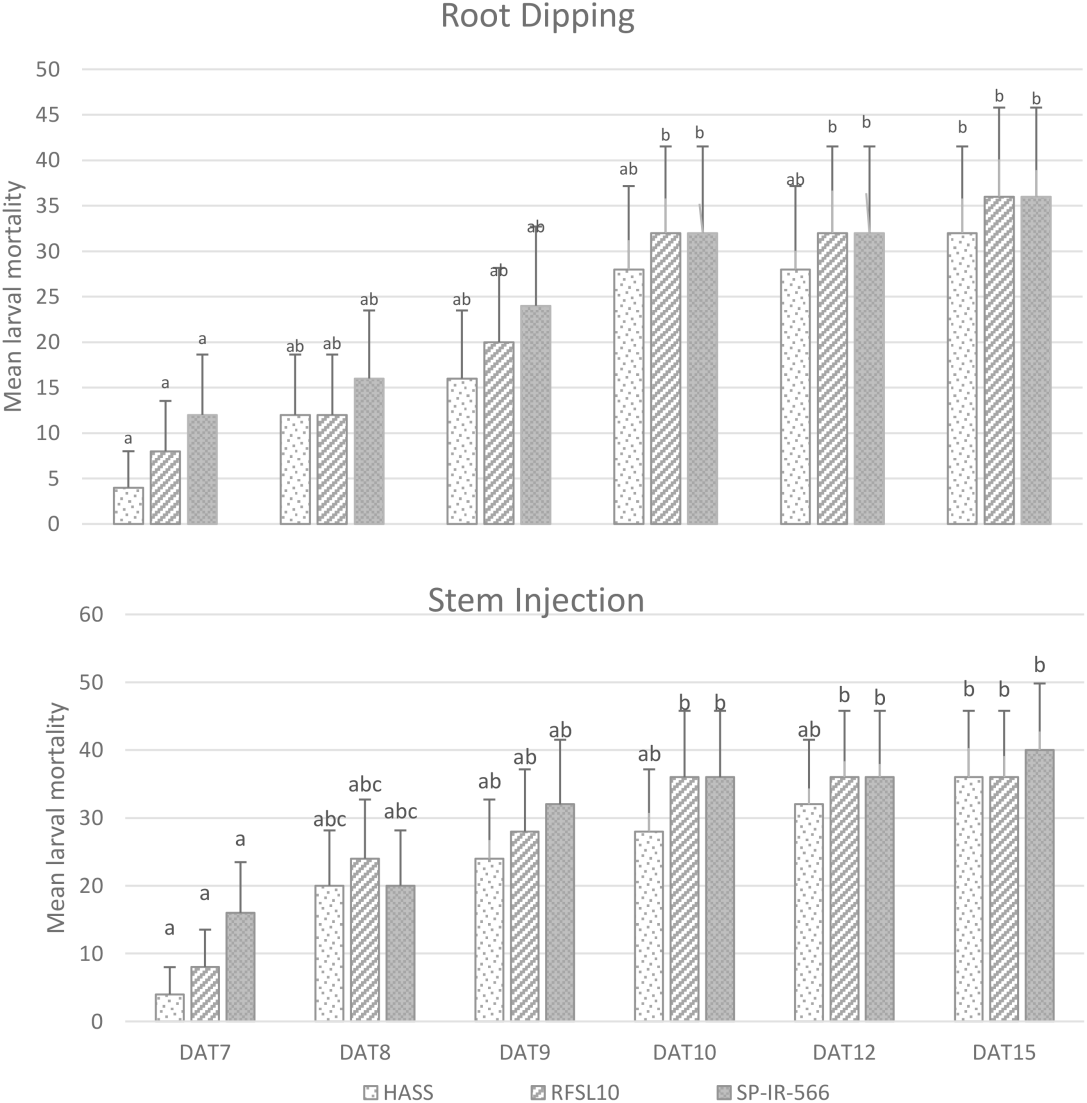
Variation of *H armigera* larvae mortality at different inoculation methods of *B bassiana* strains (HASS, RFSL10, and SP-IR-566) at 10^8^ conidia mL^-1^ under greenhouse conditions. DAT, days after treatment. The means that are followed by the different lower-case letters indicate a significant difference among different treatment time (p <0.01).

Results (**Figures 2**, **3**) demonstrated that *B. bassiana* strains were effectively established as endophytes in chickpea tissues, especially after being inoculated in the two methods: Stem Injection (SI) and Root Dipping (RD). All three strains of *B. bassiana* at 10^8^ conidia mL^−1^ were pathogenic to *H. armigera* larvae, and the percentage of larval mortality showed clear endophytic method-dependent as presented in **Figure 2**. The pathogenicity of *B. bassiana* was significant compared with the control (*p* < 0.01), and larval mortality started 5 days after treatment. The percent larval mortality ranged from 24 to 40% for different strains, 15 days after treatment. The highest percentage of mortality was obtained by the injection method, with SP-IR-566 strain reaching 40%, while HASS and RFSL10 strains achieved 36%, 15 days after treatment (**Figures 2**, **3**).

#### 3.2.2 Mean lethal time of *B. bassiana* strains

**Table 3** shows the LT_50_ recorded for each strain, based on the daily observation of *H. armigera* mortality. Using different inoculation methods, RFSL10, SP-IR-566, and HASS, strains were effective, and the LT_50_ was 10 days for RFSL and SP-IR-566 10., and 11 days for HASS, applied using stem injection method. For the root dipping method, the LT_50_ of RFSL10, SP-IR-566, and HASS was 11 days. Whereas no dead larvae were observed in the check group.

**Table 3:** Effective mean lethal time (LT_50_) and confidence limit values of three *B. bassiana* strains (HASS, RFSL10, and SP-IR-566), using different inoculation methods, against *H. armigera* larvae under greenhouse conditions.

*B. bassiana* strains	Inoculation method	LT_50_	95% confidence limit		Coefficient of determination (R²)	Graphing linear equations
			Lower limit	Upper limit		
*HASS*	SI	11.0711	9.4834	15.3801	R² = 0.9797	y = 0.104x − 0.02
	RD	11.7373	9.9193	17.4380	R² = 0.8895	y = 0.096x − 0.04
*RFSL10*	SI	10.5348	9.0923	14.0534	R² = 0.9797	y = 0.156x − 0.04
	RD	11.1176	9.5310	15.4334	R² = 0.9657	y = 0.124x − 0.02
*SP-IR-566*	SI	10.3423	8.9221	13.6840	R² = 0.9143	y = 0.132x + 0.04
	RD	11.1902	9.4769	15.8804	R² = 0.9945	y = 0.108x + 4E-16

SI, stem injection; RD, root dipping.

*LT50*: Lethal time for 50% mortality. *R* ^2^: Coefficient of determination.

### 3.3 Field evaluations

**Figure 4:**
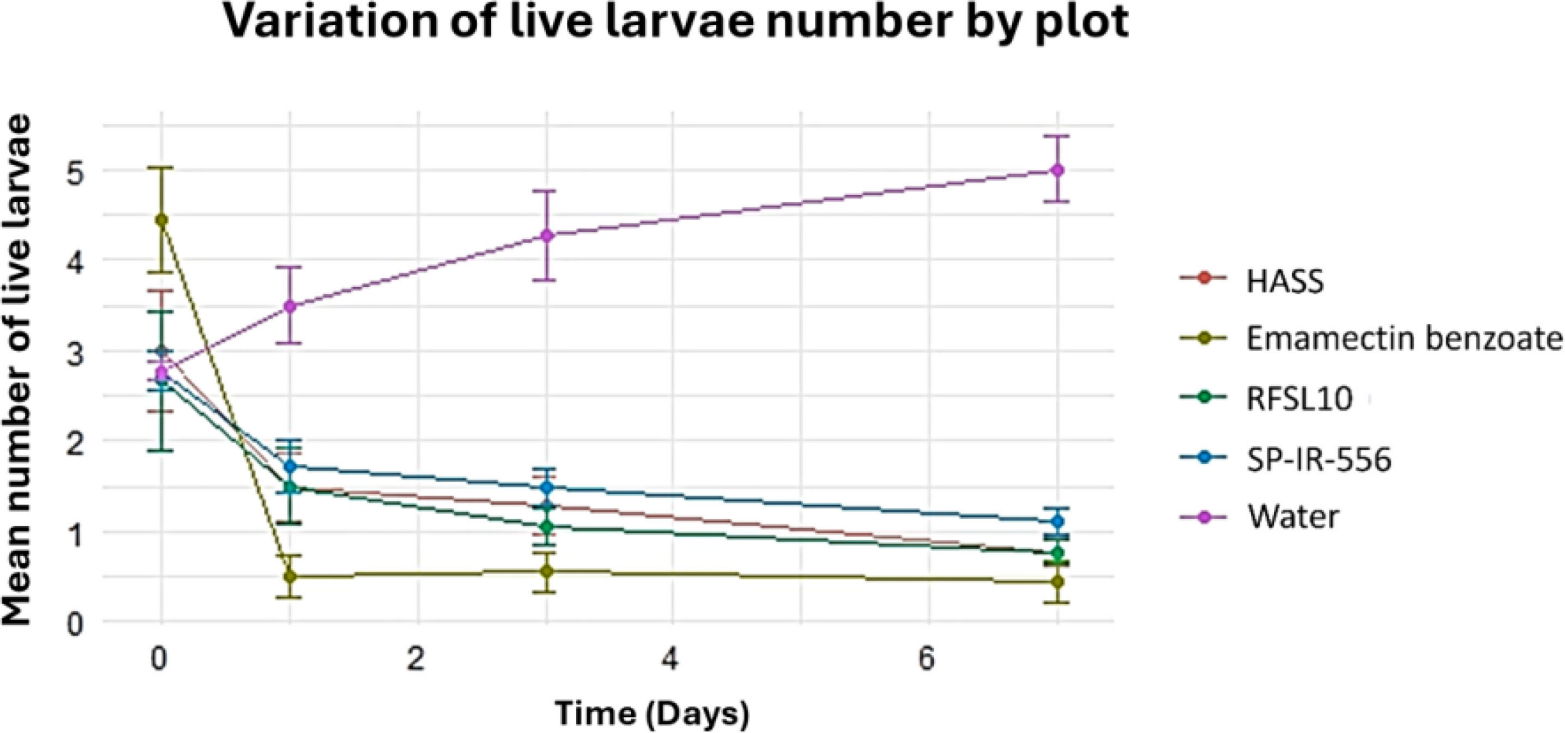
The effect of different strains and controls on the number of live larvae days after application under field conditions.

The ANOVA analysis showed a statistically significant difference in mortality of *H. armigera.* The difference in the number of live larvae between the treatments, and the control was highly significant (*p <*0.001) after 3 days of the first application of treatments. The lowest number of larvae with emamectin benzoate (0.89 larvae) was recorded one day after treatment, while the highest number of larvae was recorded in the control (2.67 larvae) on the same day after treatment, followed by *B. bassiana* strains (**Figure 4**). All tested treatments were almost equally effective in reducing the number of *H. armigera* six days after the first application (**Figure 4**).

At the end of the experiment, the results showed that the second spray seemed to differ significantly (p<0.001) in the number of live larvae between the untreated control and all studied treatments. All the evaluated treatments were comparable in their capability to reduce *H. armigera* larval population.

**Figure 5:**
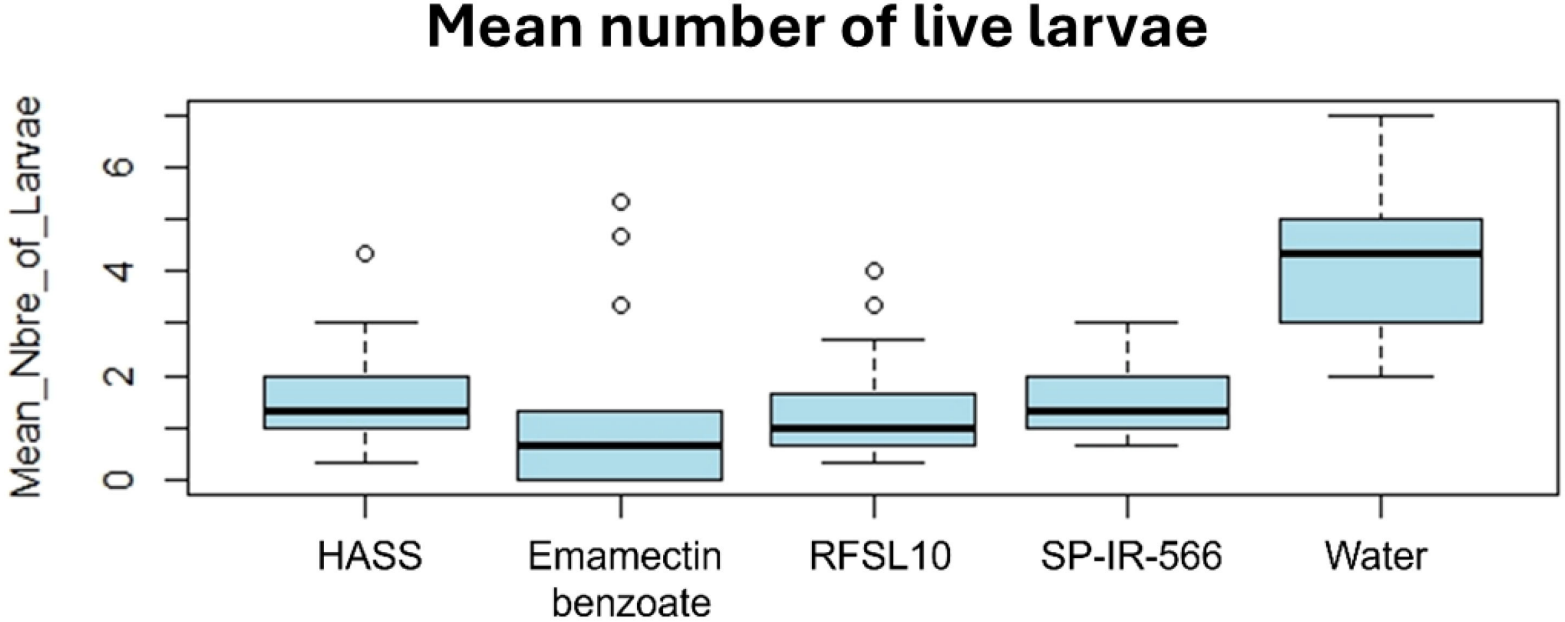
Boxplot of the toxic effect of different strains on the development of *H. armigera* larvae in comparison to the positive control (emamectin benzoate) under field conditions.

The application of emamectin benzoate reduced the number of live larvae from 3 larvae before starting the treatment to 0 larvae, seven days after the second treatment. Similarly, the strains HASS and RFSL10 reduced the number of live larvae from 3 and 2 larvae before the treatment to 0.5 larvae, one week after the second treatment. The highest reduction rate was reached by emamectin benzoate with 100%, followed by strains HASS and RFSL10 with 89 and 88%, respectively. Conversely, in the control group (water), live larvae increased from 2 to 5, 14 days after treatment (**Figure 5**).

## 4 Discussion

The present research evaluated the potential of three strains of *B. bassiana* in controlling *H. armigera* larvae in the laboratory, greenhouse, and field conditions. The current study demonstrated that all *B. bassiana* strains (RFSL10, SP-IR-566, and HASS), tested at a concentration of 10^8^ conidia mL^-1^, provided the highest toxicity on *H. armigera* using mostly direct contact and low to moderate efficacy using endophytic effect. The larval mortality and LT_50_ of the larvae increased with the increase in fungal concentrations (**Figure 1**; **Table 2**). This result is in coordination with the results of Kalvnadi (37) et al., who reported that *B. bassiana* strain (DC2) reduced the population of the second-instar larvae of *H. armigera*. Also, Petlamul et al. (38) observed that *B. bassiana*, applied at a relatively low dose, showed a remarkable efficacy against *H. armigera* larvae, and this efficacy increased as the spore concentration increased to 10^10^ conidia mL^-1^, resulting in 100% mortality. Similarly, a study conducted by Fite et al. (39) confirmed that three strains of *B. bassiana* (DLCO-EA-56, APPRC-T5, and APPRC-9604) at a concentration of 10^8^ conidia mL^-1^ were effective against the 3^rd^ instar larvae of *H. armigera* under laboratory conditions. Moreover, according to Swathi (40) et al., *B. bassiana* strain-4 caused the highest mortality rate (100%) of second instar *H. armigera*, five days after treatment. On the other hand, other studies have demonstrated the effectiveness of *B. bassiana* in controlling several lepidopteran species such as the small cabbage white butterfly *Pieris rapae* (L.) (Lepidoptera: Pieridae) (41), the diamondback moth *Plutella xylostella* (L.) (Lepidoptera: Plutellidae) (42), and the fall armyworm *Spodoptera frugiperda* (J. E. Smith) (Lepidoptera: Noctuidae) (19, 43).

In fact, according to our results, *H. armigera* was proven to be susceptible to *B. Bassiana* strains isolated not only from *H. armigera*, but also to other strains isolated from different insect species (**Table 1**). These results align with the findings reported by Suryanarayanan et al. (44) and Uma Devi et al. (45), who showed that *B. bassiana* was a generalist fungus with no strict host preference.

Furthermore, *B. bassiana* is likely to be capable of colonizing many other agronomically important crop plants. This study reported the effect of endophytic colonization of chickpea by *B. bassiana* on the survival of *H. armigera* larvae, through two different inoculation methods (SI and RD). These findings are consistent with those of Ramakuwela et al. (46), who showed that endophytic *B. bassiana* can be established in the roots, leaves, and stems of pecan seedlings (*Carya illinoinensis*), and this capacity may be used to manage pecan pests. The study found that larval mortality and LT50 increased with higher fungal concentrations, while the endophytic effect of B. bassiana on H. armigera larvae was lower with the two inoculation methods compared to direct application. This observation could be explained by the indirect effect of this fungus on chickpea by stimulating the production of secondary metabolites and/or inducing a systemic response (ISR) in the plant (47, 48), which could result in larval antifeedant behavior. Indeed, the capability of *B. bassiana* to act as an endophyte within several host plants and different plant parts was reported in corn *Zea mays* (49), tomato (50), cocoa (51), cotton (52), and opium poppy (53). Consistent with our results, Qayyum et al. (54) showed that tomato seedlings may be endophytically colonized by *B. bassiana* isolate WG-40. In addition, *B. bassiana* may protect soybean leaves by decreasing plant consumption by *Helicoverpa gelotopoeon* (Lepidoptera: Noctuidae) (55). It has been shown that *B. bassiana* occurs as an endophyte in maize plants, providing multiple benefits, including negative effects on *S. frugiperda* and *Rachiplusia nu* (Lepidoptera: Noctuidae). Additionally, it functions as a potential protective agent to control *S. frugiperda* (55, 56).

Several studies reported that this beneficial association has no negative effects on the plant’s development or physiological processes (51, 57). Numerous findings have documented the role of endophytic *B. bassiana* in promoting the growth of plant biomass. For instance, Jaber and Enkerli (58) showed that *B. bassiana*, when used as a seed dressing, may systemically colonize several plant parts and enhance plant growth. Furthermore, *B. bassiana* was able to proliferate and survive in plant tissues after being inoculated into wheat utilizing seed dressing and soil treatment techniques. This supported increased spike production in wheat plants and successfully prevented cotton leafworm (*Spodoptera littoralis*) infestation (59). The successful re-isolation of the inoculated fungal mycelia from chickpea plants confirmed the success of the colonization trial. However, the used inoculation methods expressed low to moderate mortality rates against *H. armigera*. However, there is still a lack of solid knowledge about the preferred localization of the strains within plant tissues and other factors that could enhance their performance such as using different types of genetic material.

## 5 Conclusion

The present bioassays revealed promising results for using *B. bassiana* strains (HASS, RFSL10, and SP-IR-566) to effectively control the larvae of *H. armigera*, mostly when applied as a direct spray. Using various inoculation techniques, *B. bassiana* strains were able to colonize chickpea plant tissues and reduce *H. armigera* larvae numbers. These findings show that the use of entomopathogenic fungi, either as biopesticide or endophytic, could be incorporated in the management package for the control of *H. armigera* as a safe alternative to chemical insecticides. Nevertheless, further research is needed to refine fungal formulations, understand the underlying mechanisms, and assess their compatibility with other biopesticides, such as botanical extracts or oils. These complementary studies will contribute to the establishment of a comprehensive and effective pest management program against *H. armigera.*

## Data Availability

The raw data supporting the conclusions of this article will be made available by the authors, without undue reservation.
